# An evaluation of cascading mentorship as advocacy training in undergraduate medical education

**DOI:** 10.1186/s12909-021-02489-y

**Published:** 2021-01-21

**Authors:** Mitesh Patel, Devon Aitken, Yunlin Xue, Sanjeev Sockalingam, Alexander Simpson

**Affiliations:** 1 Department of Psychiatry, Faculty of Medicine, University of Toronto, Toronto, Ontario Canada; 2 Center for Addictions and Mental Health (CAMH), 1001 Queen Street West, Toronto, Ontario M6J 1H4 Canada; 3 Faculty of Medicine, University of Toronto, Toronto, Ontario Canada

**Keywords:** Cascading mentorship, Social determinants of health, Advocacy, Undergraduate medical education

## Abstract

**Background:**

Physicians are in a position of great influence to advocate for health equity. As such, it is important for physicians-in-training to develop the knowledge and skills necessary to fulfil this role. Although various undergraduate medical programs have implemented health advocacy training, they often lack experiential learning and physician involvement. These aspects are foundational to the Advocacy Mentorship Initiative (AMI) which utilizes cascading mentorship as a novel approach to advocacy training. Medical students develop advocacy competency as peer mentors to youth raised in at-risk environments, while also being mentored themselves by physician residents. We aim to determine whether there are specific advantages to utilizing cascading mentorship to facilitate the attainment of advocacy competencies in undergraduate medical education.

**Methods:**

Medical students participating in AMI between 2017 to 2020 completed pre- and post-exposure questionnaires. Questionnaires assessed confidence in advocacy-related skills and knowledge of youth advocacy concepts, as well as learning goals, skills gained, benefits of AMI and resident mentors, and impact on future career. Sign tests were utilized to analyze quantitative results, and content analysis was used for open-ended responses. A triangulation protocol was also utilized.

**Results:**

Fifty mentors participated, 24 (48%) of which completed both pre- and post-exposure questionnaires. Participants gained confidence in advocacy-related skills (*p* < 0.05) such as working with vulnerable populations and advocating for medical and non-medical needs. They also reported significant improvements (*p* < 0.01) in their understanding of social determinants of health and concepts related to children’s health and development. Content analysis showed that participants built meaningful relationships with mentees in which they learned about social determinants of health, youth advocacy, and developed various advocacy-related skills. Participants greatly valued mentorship by residents, identifying benefits such as support and advice regarding relations with at-risk youth, and career mentorship. AMI impacted participants’ career trajectories in terms of interest in working with youth, psychiatry, and advocacy.

**Conclusions:**

AMI offers a unique method of advocacy training through cascading mentorship that engages medical students both as mentors to at-risk youth and mentees to resident physicians. Through cascading mentorship, medical students advance in their advocacy-related skills and understanding of social determinants of health.

**Supplementary Information:**

The online version contains supplementary material available at 10.1186/s12909-021-02489-y.

## Background

The role of a physician as Health Advocate is a central pillar in the development of competencies deemed necessary to practice medicine effectively, as described by the CanMEDS framework [1]. The CanMEDS framework has been adopted by medical programs in dozens of countries [2]; while other countries have included advocacy to established competency frameworks (for example, the Accreditation Council for Graduate Medical Education in the United States has included advocacy in the Core Competency of “Systems-based Practice” [3]). By developing skills in the Health Advocate role, medical students learn to identify and address inequities in the social determinants of health and the manner in which these influence health outcomes in various populations [4]. Importantly, preclinical advocacy training provides the unique opportunity for students to develop their identity as advocates, unrestricted by the realities of clinical responsibilities that can overshadow patient care [5]. Nevertheless, advocacy training has historically been an underappreciated component of medical training [6]. The effective integration of advocacy training in undergraduate medical education presents various challenges [7], including the difficulty to teach and evaluate advocacy [8].

Experiential learning and community engagement in health advocacy training has been implemented in various undergraduate medical education programs [9]. Several medical schools, including the University of Toronto (UofT), have integrated community-based service learning (CBSL) as a tool for medical trainees to engage in experiential learning opportunities while also addressing community needs [10]. CBSL has been described as an effective method to teach social determinants of health [11] and provides trainees with the opportunity to directly observe health inequities [12, 13]. Appreciating social determinants of health and health inequity are foundational to engage in health advocacy [4].

One example of CBSL involves mentoring youth raised in at-risk environments. Various studies have shown significant positive impact upon mentees [14], particularly for at-risk youth [15]. Furthermore, mentors gain valuable skills and insights, which can extend to areas pertaining to effective advocacy training. One study found that through mentoring at-risk youth, university students had an increased sense of civic responsibility, and a belief that “it is every person’s responsibility to use their time and talents to help solve social problems” [16]. A thematic analysis of medical students’ reflections while mentoring youth from a Native Hawaiian community found that the experience developed their skills in establishing relationships, self-reflection, communication, compassion, as well as led to a greater understanding of health inequity [17]. Literature pertaining to the extent to which mentoring may serve as an opportunity to develop advocacy skills and competencies remains relatively lacking, especially in the context of medical education.

Medical students are also often mentees in mentoring relationships with senior medical trainees and physicians. A review of such mentorship programs identified benefits for medical student mentees in clinical knowledge and skills attainment, professional development, communication skills development, exposure to subspecialties, career guidance, and opportunities for networking and research involvement [18]. However, development of health advocacy competencies in medical students through physician mentorship remains a poorly described area of study. Recognizing this gap, Luft [19] calls upon medical schools to utilize physician advocates as mentors, teachers, and role models for medical trainees to develop advocacy skills, especially within a formal curriculum.

**Fig. 1:**
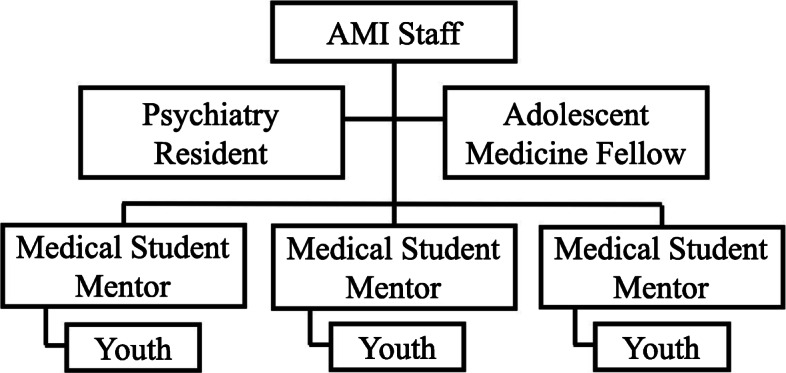
AMI Mentoring Group. The structure of the Advocacy Mentorship Initiative (AMI) program is one of cascading mentorship where the 3 or 4 medical students (mentors) are mentors to at-risk youth (mentees), and are in turn mentored by a Psychiatry resident and Adolescent Medicine fellow (residents), forming a mentoring group. The AMI staff (the primary author a Child and Adolescent Forensic Psychiatrist and Peer Project staff) oversees all mentoring groups and provides additional guidance to the residents and medical students

Here we describe and evaluate an innovative advocacy training program, called the Advocacy Mentorship Initiative (AMI). AMI utilizes a cascading mentorship model that enables preclinical medical students to become peer mentors for youth raised in at-risk environments, while also receiving mentorship from postgraduate medical trainees (resident physicians) (see Fig. 1). The objectives of AMI, for medical students specifically, are to foster advocacy skills and improve their understanding of issues facing at-risk youth, child and adolescent development, and social determinants of health. We aim to determine the benefits of utilizing a cascading mentorship model to facilitate the attainment of advocacy skills in undergraduate medical education by elucidating medical student mentor experiences through questionnaires that elicit learning goals, evaluate self-confidence in advocacy-related skills and knowledge, and provide the opportunity for students to describe the befits of participation, career impact, and experience of having a resident mentor.

## Methods

### Program description: the advocacy mentorship initiative (AMI)

The Advocacy Mentorship Initiative (AMI) is a supplemental advocacy curriculum at the UofT that has provided medical students with focused advocacy training since 2014. AMI pairs pre-clerkship medical students (herein referred to as mentors) to youth raised in at-risk environments, (herein referred to as mentees). The mentees are identified as having significant emotional, social, and/or behavioral concerns, which are implicitly connected to their social determinants of health. The curriculum runs for approximately one year, starting in March of the first year of medical school and extends to completion of the second year.

AMI follows a cascading mentorship model in which mentors are provided with teaching and supervision by a UofT Psychiatry Resident and an Adolescent Medicine Fellow, (herein referred to as residents or resident mentors) in groups of three or four medical students to two residents (herein referred to as mentoring groups). The primary author, a dually qualified child and adolescent and forensic psychiatrist, provided over-arching support and supervision to all participants (see Fig. 1). Due to unforeseen circumstances, the 2019–2020 cohort did not include Adolescent Medicine mentors and thus proceeded with Psychiatry Residents only.

All mentors and residents met together monthly for large group supervision and teaching related to advocacy. Flipped-classroom teaching methods were utilized. The large group sessions were overseen by the primary author. Mentors and residents provided updates regarding pertinent matters related to the matches through facilitated group discussion. Confidentiality of the mentees was preserved; other mentors, residents and staff were not made aware of the full names of mentees. Each resident also provided at least one 45-min didactic teaching session during these monthly meetings. The sessions were facilitated by e-modules [20] and evaluations were completed by the entire group following each of the sessions. Social justice, humanism, and critical consciousness informed the topics discussed, as well as principles related to child development and advocacy.

In addition, each mentoring group met on a biweekly basis and residents provided their assigned mentors with supervision and mentorship to foster the mentor’s peer relationship with their youth and to discuss various social determinants of health.

Mentors completed mandatory CBSL training or Enriching Educational Experiences requirements, thus fulfilling curricular requirements related to their medical doctoral degrees. Residents completed evaluations and provided summative feedback at the completion of the program. Mentors were required to attend at least 75% of the teaching sessions to obtain a letter of completion with comments provided by their residents. This letter contributed to their undergraduate medical education portfolio. Residents were also provided with letters of completion outlining their involvement in the program. Evaluations and comments from mentors assigned to each resident were included in these letters, which were also added to their postgraduate training portfolios.

Mentors were engaged in AMI as registered volunteers of The Peer Project, a community-based organization (CBO), which matches vulnerable youth aged 6–15 to young adult mentors between the ages of 16–29. The Peer Project is a non-denominational and non-profit organization supported by the United Way, a national non-governmental organization. Youth who were deemed to be raised in at-risk environments were matched to mentors in AMI. Staff members from the Peer Project were included in monthly meetings to inform discussions from a CBO perspective.

### Participants

Ethics approval was obtained from the UofT Research Ethics Board. Inclusion criteria consisted of active enrollment in the UofT medical school and AMI program, and informed written consent was obtained to participate (see Table 1). Medical students were enrolled in March of their first year of undergraduate medical education in 2017, 2018, 2019, and 2020. All 62 mentors from these cohorts were invited to participate. Due to the timing of data collection, only mentors from cohorts 2018–2019 and 2019–2020 were invited to participate in both the pre- and post-exposure questionnaires.

**Table 1:** Population, intervention, comparison, and outcome information pertaining to this study

POPULATION	Second year medical students at UofT enrolled in AMI who provided informed consent to participate in the AMI program.
INTERVENTION	Weekly 1.5 h peer mentorship, 2 h monthly discussions with psychiatry and adolescent medicine residents, and 1 h monthly large group teaching sessions.
COMPARISON	Pre-exposure knowledge of youth mental and physical health topics and confidence levels in skills vs. post-exposure knowledge and skill levels.
OUTCOME	Unique experiential learning opportunity to gain advocacy skills.

### Recruitment of medical students (mentors)

The primary author recruited students through a lunch presentation in January of the first year for each of the cohorts: 2017–2018, 2018–2019, 2019–2020, and 2020–2021. Students submitted applications for the program and all students who applied were accepted. Participants attended a full-day training session coordinated by the Peer Project in March of each year regarding regulations, policies, and mentorship techniques. In April of each year, The Peer Project began the match process between mentors and mentees. Matches began to engage in activities once pairings were complete with an aim to meet for an average for 1.5 h per week. Structured teaching and group sessions began in September of each year and ended in May of the following year (9 months total). Matches could continue to engage through ongoing enrollment with the Peer Project after May of each year.

### Recruitment of residents

A recruitment e-mail was sent to Psychiatry residents in years two to five of a five-year training program and to Adolescent Medicine fellows (fifth year of pediatric training) in July of 2017, 2018, 2019, and 2020. Those interested responded directly to the e-mail and respondents were enrolled until all positions were filled. Resident mentors were advised of their mentoring groups by August of each year and began meeting in their groups until completion of the program in May.

### Data collection

Data was collected through pre- and post-exposure questionnaires (see Additional File 1). Questionnaires were sent to medical student mentors via e-mail prior to their match with their mentees and following completion of the AMI program. Questionnaires included both Likert scales and short-answer questions. Each participant was assigned a unique and anonymous identifier by an administrative assistant; the investigators remained blinded to these codes. The questionnaire asked participants to rate their level of knowledge and skills regarding various topics and their level of confidence regarding skills on a five-point Likert Scale (1 = Poor, 2 = Fair, 3 = Good, 4 = Very good, 5 = Excellent). The pre-exposure questionnaire also included open-ended questions asking students to list their learning goals. In the post-exposure questionnaire, they were asked open-ended questions regarding skill development, benefit of involvement in AMI and resident mentors, and the impact of AMI on their career path.

### Data analysis

Questionnaire items using Likert scales were scored 1 to 5 (1 = Poor, 5 = Excellent). The distribution of differences between the pre- and post-exposure data was neither normal nor symmetrical; thus, sign tests were used to compare the median differences. Pre- and post-exposure open-ended questionnaire responses were analyzed using content analysis, employing the approach described by Taylor-Powell and Renner [21]. Upon reading through the responses numerous times, the data were categorized as themes emerged to develop a coding scheme. Categories were constructed until no new themes or subcategories were identified. The responses were again reviewed with the completed coding scheme. Utilizing the identified codes, the data was sorted into themes relevant to the focus of the study and any connections between themes were identified.

A triangulation protocol was adapted from Tonkin-Crine and colleagues [22] to integrate Likert scale and open-ended responses regarding communication skills. Pairwise comparisons were made between the data sets to identify convergence. Pairwise comparisons were considered in “Dissonance” if the participant described that they had gained communication skills but did not demonstrate an increase in confidence in their communication skills by quantitative measures. Pairwise comparisons were considered in “Agreement” if the participant described that they had gained communication skills, as well as demonstrated an increase in confidence in their communication skills by quantitative measures. Pairwise comparisons were considered in “Silence” if the participant demonstrated increased confidence in communication skills but did not describe communication skills as skills gained during open-ended responses.

## Results

A total of 50 (out of 62 mentors contacted) participated in the study by completing at least one questionnaire (pre- or post-exposure), resulting in a response rate of 50/62 (81%). Thirty-six mentors completed the pre-exposure questionnaire (response rate of 36/50 or 72%), while 38 mentors completed the post-exposure questionnaire (response rate of 38/42 or 90%). Thirty mentors (from cohorts 2018–2019 and 2019–2020) were invited to participate in both the pre- and post-exposure questionnaires, resulting in a response rate of 24/30 (80%). Out of the 50 participants, 39 were female (78%). The mean age of participants was 23.64 (SD 1.21) and the mean age for mentees was 11.51 (SD 2.48). The mean duration of the matches was 9.07 months (SD 2.61).

### Pre-exposure self-identified goals

The pre-exposure questionnaire asked mentors (*n* = 36) to list up to four objectives they wish to achieve prior to starting the AMI program. Twelve themes emerged, as summarized in Table 2. The most common goal identified was to gain a better understanding of psychiatric and developmental disorders in youth. Other goals included: improve communication skills with youth and families, learn how to advocate, build relationships with youth, increased comfort working with youth, have a positive impact on mentee, become aware of community resources, contribute to the community, career development and exploration, as well as to gain a better understanding of social determinants of health, child development, and physical illness in youth.

**Table 2:** Medical student mentors’ goals for the AMI program

Goals	Number ***n*** = 36	Representative Quotations
Gain a better understanding of psychiatric and developmental disorders in youth	18	“Better understanding of psychiatric and developmental disorders in youth”
Improve communication skills with youth and families	16	“Develop skills in speaking with the child and their family”
Build relationships with youth	12	“create a reliable and supportive relationship with my mentee”
Gain a better understanding of social determinants of health	11	“Ability to appreciate the effects that...social determinants of health may have on a child and their family.”
Career development and exploration	11	“career development and insight from residents/fellows/staff”; “Evaluate my interest in pediatrics and psychiatry”
Learn how to advocate	7	“Gain skills in advocating for the needs of at-risk youth and/or young patients with mental illness”
Gain a better understanding of child development	7	“Understand the needs for healthy psychological, emotional and social development for youth and how to facilitate that”
Gain a better understanding of physical illness in youth	5	“Learn about physical illnesses in children”
Increased comfort working with youth	5	“increased comfort interacting with children”
Have a positive impact on mentee	5	“I hope I would have made a positive impact, however small, on my junior.”
Become aware of community resources	3	“Learn more about services and opportunities available for people living in Toronto”
Contribute to the community	2	“Contribute and give back to the community”

### Self-reported confidence levels

Confidence levels pertaining to 8 skills associated with the AMI program were compared and summarized in Table 3. A sign test demonstrated significant increases (*p* < 0.05) in respondents’ (*n* = 24) confidence regarding working with vulnerable populations and advocating for medical and non-medical needs. There was no significant effect on confidence regarding communicating with youth, their family members, or with staff who provide care to youth. There was no significant effect on confidence involving working with children who have mental illness or a chronic medical illness.

**Table 3:** Self-reported level of confidence on various tasks before and after the AMI program

Task	Pre-Exposure			Post-Exposure			
	Mean	Median	SD	Mean	Median	SD	***p***-value
Communicating with youth	3.25	3.00	0.676	3.29	3.00	0.550	1.000
Communicating with patient’s family member	2.83	3.00	0.637	3.00	3.00	0.780	0.454
Communicating with staff who are in a supervisory or care provider role of the youth	2.83	3.00	0.868	2.83	3.00	0.816	1.000
Working with vulnerable populations	2.54	3.00	0.833	2.88	3.00	0.680	0.039*‡*
Working with children who have mental illness	2.43	2.00	1.037	2.39	2.00	0.839	1.000
Working with children who have a chronic medical illness	2.13	2.00	0.947	2.13	2.00	0.741	0.754
Advocating for the medical needs of your patient	2.29	2.00	0.908	2.75	3.00	0.608	0.022*‡*
Advocating for the non-medical needs of your patient	2.29	2.00	0.955	2.67	3.00	0.761	0.013*‡*

*‡p < 0.05.*

### Self-reported knowledge levels

Level of knowledge pertaining to five domains were compared and summarized in Table 4. Sign tests demonstrated statistically significant increases in participants’ (*n* = 24) self-rating of their level of knowledge across all five domains of knowledge (*p* < 0.001). Specifically, there was a statistically significant median increase in social determinants of health (1.00), child development (1.50), attachment theory (2.00), chronic illness in youth (1.00) and autism and other developmental disabilities (1.50).

**Table 4:** Self-reported level of knowledge before and after the AMI program

Domain	Pre-Exposure		Post-Exposure		
	Median	SD	Median	SD	p-value
Social determinants of health	3.00	0.859	4.00	0.537	< 0.01
Child development	2.00	0.721	3.50	0.824	< 0.01
Attachment theory	2.00	0.830	4.00	1.062	< 0.01
Chronic illness in youth	2.00	0.584	3.00	1.083	< 0.01
Autism and other developmental disabilities	2.00	1.007	3.50	0.881	< 0.01

### Self-reported skills gained

Thirty-one of 38 respondents (82%) answered “Yes, I have gained new skills.” Identified themes included skills in communication, relationship building with youth, advocacy, finding community resources, cultural competency, and understanding social determinants of health. Data are summarized in Table 5, including direct quotations taken from answers pertaining to each theme.

**Table 5:** Skills gained through the AMI program as described by medical student mentors

Skills Gained	Number ***n*** = 29	Representative Quotations
Communication	21	“Learning how to adapt, effectively connect and communicate with a youth from vulnerable population and their supports.”
Relationship building with youth	10	“Building a relationship and gaining trust of an adolescent.”
Advocacy	5	“Advocating on behalf of youth and their families”
Finding community resources	3	“Ability to search for youth-friendly and cost-effective activities and resources available in a community.”
Cultural competency	2	“Understanding cultural practices”
Understanding social determinants of health	2	“We learn about [social determinants of health], but to go into the environment and hear first-hand the experiences of my youth really helped me to understand these determinants.”

### Self-reported benefits of AMI

Respondents (*n* = 32) identified benefits related to their engagement with AMI. From the textual answers provided, we identified 7 themes. The main benefit of the AMI program was identified as building a relationship with a mentee. Other themes that emerged included building a relationship with the resident mentors, positive impact on mentee, learning about child development concepts and child psychiatry, learning about real-life challenges first-hand, improving communication skills, and learning to advocate for youth. Table 6 provides representative quotations taken from responses related to the identified themes.

**Table 6:** Benefits of the AMI program as described by medical student mentors

Benefits of AMI	Number ***n*** = 32	Representative Quotations
Building a relationship with the mentee	18	“The relationship I was able to form with my mentee and be reminded of the reasons I went into medical school.”
Building a relationship with the resident mentors	6	“the relationships I formed with the resident mentors and the guidance and support they offered.”
Positive impact on mentee	6	“The opportunity to make a real impact on a youth from a vulnerable background”
Learning about child development concepts and child psychiatry	6	“The combination of learning concepts and theory in child and adolescent psychology and then immediately witnessing and applying it towards the relationship with my mentee was highly effective in reinforcing these concepts.”
Learning about real-life challenges first-hand	4	“It is a completely different experience to learn about social determinants of health vs seeing for yourself how much those SDOHs [social determinants of health] really impact daily life of a family.”
Improving communication skills	3	“increased experience in communicating with … pediatric populations”
Learning to advocate for youth	2	“Understanding how to advocate for youth living in at-risk populations.”

### Impact on future career

Twenty-three of 38 (61%) respondents indicated that participation in the AMI program influenced their future career. When asked to elaborate, textual responses provided codes generating five themes. The most common theme was that AMI strengthened mentors’ interest in working with youth. Other themes included awareness of social determinants of health, greater interest in psychiatry, greater interest in advocacy, and a better understanding of the advocacy role. Table 7 provides direct quotations taken from responses related to each of the themes.

**Table 7:** Impact of AMI on career trajectory as described by medical student mentors

Impact on Career Trajectory	Number ***n*** = 23	Representative Quotations
More interest in working with youth	11	“This has reinforced my desire to work with youth as I see how important this period is developmentally and the opportunity to change the life trajectory of people in your care”
Awareness of social determinants of health	6	“I will now take into account social determinants of health when I recommend treatment plans for patients and their families.”
More interest in psychiatry	5	“Furthered my interest in psychiatry”
More interest in advocacy	3	“It just re-affirmed that I want to participate a lot in advocacy in whatever speciality I end up in.”
Better understanding of advocacy role	3	“Better understanding of how community programs and advocacy on the individual and organizational level play a role in child health and development”

### Benefits of resident mentors

Thirty-five of 38 (92%) participants listed benefits from engaging with their resident mentors. From these responses, four themes were identified. The most common themes identified were that residents provided general advice and support as well as provided advice on handling difficult situations. Other themes included career mentorship and promoted discussion regarding mentorship experiences. These themes and sample responses are provided in Table 8.

**Table 8:** Benefits of having resident mentors as described by medical student mentors

Benefits of Resident Mentors	Number ***n*** = 35	Representative Quotations
General advice and support	18	“They always took the time to check in with us and provide feedback on our situations with our mentees using their knowledge from paediatrics and psychiatry. This was helpful connecting my experience to the curriculum. They also took the time to understand our matches and talk about our emotions.”
Advice on handling difficult situations	18	“The advice they gave and the ability to talk about any problems we had and brainstorm ways to approach difficult or challenging situations with our mentees”
Career mentorship	7	“They went beyond what was required and helped support us both in the program and with our life circumstances and career aspirations.”
Promote discussion regarding mentorship experiences	4	“The supervising residents were able to mediate and promote highly constructive discussion amongst my classmates surrounding recurring themes.”

### Interpretive analysis of communication skills

Of 38 findings from 24 participants, there were 7 instances of agreement, 13 instances of dissonance, and 18 instances of silence.

#### Instances of agreement

Five participants demonstrated an increase in confidence in their communication skills, as well as described that they gained communication skills. For one participant, they described gaining communication skills in all three categories (with youth, families, and staff who are in a supervisory or care provider role of the youth) and this was in agreement with increased confidence in these domains, resulting in a total of 7 instances of agreement.

#### Instances of dissonance

There were 13 instances (by a total of 10 participants) in which gaining communication skills with youth and/or their families were described but were not associated with an increase in confidence in that domain. For two participants, they described gaining communication skills in general, but this was not associated with an increase in confidence in any communication domain (with youth, families, and staff who are in a supervisory or care provider role of the youth).

#### Instances of silence

There were 18 instances (by a total of 11 participants) in which there was an increase in confidence in communication skills with youth, families, or with staff who are in a supervisory or care provider role of the youth, but without describing this increase in communication skills during open-ended responses.

## Discussion

AMI aims to provide medical students with advocacy training through a unique cascading mentorship program. Medical students engaged with youth raised in at-risk environments and had the opportunity to learn about the broader implications of social determinants of health outside of a clinical setting. Medical students were in turn mentored by resident physicians who provided support in their relations with at-risk youth, as well as career mentorship. The knowledge and skills gained, as well as the relationships built with mentees, generated improved understanding and ability to advocate for others.

Previous research demonstrated that medical students who mentored youth from an underserved community developed confidence in skills such as establishing relationships, self-reflection, compassion, teaching, and communication, along with a better understanding of health inequity [17]. The focus of AMI is to prepare medical students to fulfil their role as Health Advocate. As health advocates, physicians are expected to work with and advocate for vulnerable populations, including youth. AMI aided mentors in gaining confidence in skills such as working with vulnerable populations and advocating for the needs of youth. Medical students also described gaining skills such as relationship building with youth, finding community resources, cultural competency, and understanding social determinants of health – all skills necessary to advocating for and with youth [4, 23]. Although “understanding social determinants of health” may be better described as knowledge, this understanding is foundational to advocacy that addresses social determinants of health. Several medical students described gaining “advocacy skills” explicitly.

CBSL allows medical students to directly observe health inequities [12, 13] and learn about social determinants of health [11]. AMI is an example of CBSL, and as such, mentors suitably identified such benefits from involvement in the program. Mentors discussed building a relationship with their mentee, learning about real-life challenges first-hand (including those related to living in poverty and other social determinants of health), and learning to advocate for youth. Additionally, mentors discussed having a positive impact on their mentee as a benefit of AMI, including teaching their mentee new things or aiding in the development of the youth’s self-confidence.

Mentors also described improved communication skills as a benefit of participating in AMI. Being able to effectively communicate is a skill that is important for building relationships as physicians [24], and is foundational to developing competency as a Health Advocate [25]. Interestingly, ten mentors did not demonstrate increased confidence in communication skills, as measured by pre- and post-exposure methods, but did describe gaining communication skills. Considering that confidence was highest for communication skills at baseline, it may be that medical students were over-confident in their communication skills prior to AMI. However, through participating in AMI, they were provided an opportunity to assess their communication skills with youth and families more realistically. Nonetheless, once mentors reflected on their experience with AMI, many mentors described gaining communication skills generally, with youth or with youth’s family and other supports. Objective measures of communication skills before and after participation in AMI may have provided a better determination of whether communication skills were improved.

Physician advocates have been recognized as a valuable, yet under-utilized resource for developing advocacy skills in medical students [19]. The availability of physicians as mentors in AMI is especially important considering that mentors who feel unsupported leads to termination of mentorship relationships [26]. Suitably, medical student mentors identified the relationships they built with resident mentors as a benefit of participating in AMI. Furthermore, the medical students outlined various benefits of having resident mentors. From the benefits described, it is evident that the bi-weekly meetings with resident mentors were an opportunity for the medical students to discuss their mentorship experiences among peers, as well as receive advice and support from resident mentors, especially when faced with difficult situations. These meetings were also an opportunity for career mentorship, as many programs providing mentorship to medical students tend to initiate [18].

Moreover, students learned about social determinants of health, child development, attachment theory, chronic illness in youth, and autism and other developmental disabilities. AMI provided directed teaching with mentors surrounding these five domains, as it is foundational knowledge held by various professionals that are best suited to not only understand and work with youth, but also to advocate for youth [23].

Furthermore, the majority of medical student mentors indicated that their experiences would inform their future practice. AMI promoted interest in working with youth, in the field of psychiatry, or in advocacy. Students also recognized that AMI offered an experience that taught them to be more aware of social determinants of health and gain a better understanding of the advocacy role.

The most commonly reported goals of participating in AMI was to gain a better understanding of psychiatric and developmental disorders in youth and child development more generally; improve communication skills with youth and families; build relationships with youth; gain a better understanding of social determinants of health; and learn how to advocate. Another commonly reported goal was for career development and exploration. It is evident that these goals were aligned with the purpose of AMI and were realized throughout the program.

### Limitations

Only mentors from two cohorts were invited to participate in both pre- and post-exposure questionnaires, thus limiting the ability to draw comparisons across the entire group for analyses that required longitudinal data. Furthermore, not all medical student mentors invited to participate in pre- and/or post-exposure questionnaires decided to do so. As such, it is possible that medical student mentors that participated in the study are not representative of all mentors that participated in AMI, which may bias the impacts of this program. Additionally, it is unclear how medical students that did not participate in AMI would rate their level of confidence in various skills and knowledge domains assessed in this evaluation, as they were not included in the study. Lastly, medical students were asked to self-report their level of confidence in various advocacy skills, and as such may not directly correlate with competence in those skills.

### Future directions

To our knowledge, AMI represents the only cascading mentorship program that positions medical students as both mentors to at-risk youth and mentees to resident physicians. As such, future directions for research include expanding our understanding of cascading mentorship in medical education. This will include examining how resident physicians are impacted by the program, including development of skills related to mentoring, teaching, and professionalism.

## Conclusions

AMI offers a novel approach to developing advocacy in medical students by integrating experiential community-based learning with physician engagement through a cascading mentorship model. Medical students gained confidence in their advocacy skills as well as gained a better understanding of social determinants of health, children’s health and development, and youth advocacy. AMI also led to furthered interests in careers related to working with youth, psychiatry, and advocacy. It is through both mentoring at-risk youth and being mentored by residents that medical students were provided the opportunity to develop as our future physician advocates.

## Supplementary Information

**Additional File 1.** Questionnaires. This file contains the questionnaires provided to medical student mentors prior to and following participation in the Advocacy Mentorship Initiative.

## Data Availability

The datasets used and/or analysed during the current study are available from the corresponding author on reasonable request.

## Abbreviations

UofT: University of Toronto
AMI: Advocacy Mentorship Initiative
CBSL: community-based service learning
CBO: community-based organization
