# Supplementary material for: An evaluation of cascading mentorship as advocacy training in undergraduate medical education
Source: BMC Med Educ. 2021 Jan 21;21:65. doi: 10.1186/s12909-021-02489-y (PMC7818733; doi:10.1186/s12909-021-02489-y)
Supplement: Supplementary file 1 — Additional File 1. Questionnaires. This file contains the questionnaires provided to medical student mentors prior to and following participation in the Advocacy Mentorship Initiative. [file 12909_2021_2489_MOESM1_ESM.pdf]

Pre-exposure questionnaire

From the list below, rate your level of understanding with each topic area prior to entering the AMI program**:**

	Poor	Fair	Good	Very Good	Excellent
Social determinants of health	1	2	3	4	5
Child development	1	2	3	4	5
Attachment theory	1	2	3	4	5
Chronic illness in youth	1	2	3	4	5
Autism and other developmental disabilities	1	2	3	4	5

Please rate your current level of confidence with the following tasks:

	Poor	Fair	Good	Very Good	Excellent
Communicating with youth	1	2	3	4	5
Communicating with patient's family member	1	2	3	4	5
Communicating with staff who are in a supervisory or care provider role of the youth	1	2	3	4	5
Working with vulnerable populations	1	2	3	4	5
Working with children who have mental illness	1	2	3	4	5
Working with children who have a chronic medical illness	1	2	3	4	5
Advocating for the medical needs of your patient	1	2	3	4	5
Advocating for the non-medical needs of your patient	1	2	3	4	5

What are 2 to 4 personal learning objectives that you would like to reach by the end of the AMI program?

Post-exposure questionnaire

From the list below, rate your level of understanding with each topic area:

	Poor	Fair	Good	Very Good	Excellent
Social determinants of health	1	2	3	4	5
Child development	1	2	3	4	5
Attachment theory	1	2	3	4	5
Chronic illness in youth	1	2	3	4	5
Autism and other developmental disabilities	1	2	3	4	5

Please rate your current level of confidence with the following tasks:

	Poor	Fair	Good	Very Good	Excellent
Communicating with youth	1	2	3	4	5
Communicating with patient's family member	1	2	3	4	5
Communicating with staff who are in a supervisory or care provider role of the youth	1	2	3	4	5
Working with vulnerable populations	1	2	3	4	5
Working with children who have mental illness	1	2	3	4	5
Working with children who have a chronic medical illness	1	2	3	4	5
Advocating for the medical needs of your patient	1	2	3	4	5
Advocating for the non-medical needs of your patient	1	2	3	4	5

Did you gain any new skills from your participation in the program?

- o Yes, I have gained new skills
- o I am unsure
- o No, I have not gained new skills

If yes, please specify:

What was the greatest benefit from your involvement in the AMI program?

What was most helpful about your interactions with your supervising residents?

Do you believe the experience as a mentor will influence your career trajectory or future practice?

- o Yes
- o Unsure
- o No

If yes, please describe the program's impact on your future career path:
